# 

**Published:** 1876-03

**Authors:** 


### BOUND VOLUMES.

Any one sending us $4 will receive two copies of HALL'S JOURNAL OF HEALTH for one year, and the Volume for 1874 or 1875 (beautifully bound in red cloth, red edges, and gilt letters on back), postage paid. Any one sending $6, will be entitled to three subscriptions for one year, and the bound volumes for 1874 and 1875. Either volume sent post paid for $1.50. Address, HALL'S JOURNAL OF HEALTH, 137 Eighth street, New York.

